# Maternal HIV infection is associated with distinct systemic cytokine profiles throughout pregnancy in South African women

**DOI:** 10.1038/s41598-021-89551-3

**Published:** 2021-05-12

**Authors:** Charlene Akoto, Shane A. Norris, Joris Hemelaar

**Affiliations:** 1 Nuffield Department of Women’s and Reproductive Health, University of Oxford, The Women’s Centre, John Radcliffe Hospital, Oxford, UK; 2 South African Medical Research Council Developmental Pathways for Health Research Unit, Department of Paediatrics, School of Clinical Medicine, University of the Witwatersrand, Johannesburg, South Africa; 3 National Perinatal Epidemiology Unit, Nuffield Department of Population Health, University of Oxford, Richard Doll Building, Old Road Campus, Oxford, OX3 7LF UK

**Keywords:** Cytokines, HIV infections

## Abstract

Maternal HIV infection is associated with adverse pregnancy outcomes, but the mechanisms remain unknown. The course of pregnancy is regulated by immunological processes and HIV infection and antiretroviral therapy (ART) impact key immune mechanisms, which may disrupt the immune programme of pregnancy. We evaluated a broad range of systemic cytokines at each trimester of pregnancy in 56 women living with HIV (WLHIV) and 68 HIV-negative women, who were enrolled in a prospective pregnancy cohort study in Soweto, South Africa. The pro-inflammatory cytokine IP-10 was detected in each trimester in all WLHIV, which was significantly more than in HIV-negative women. The anti-viral cytokine IFNλ1 was detected more frequently in WLHIV, whereas IFNβ and IFNλ2/3 were detected more frequently in HIV-negative women. Th1 cytokines IL-12 and IL-12p70, Th2 cytokine IL-5, and Th17 cytokine IL-17A were detected more frequently in WLHIV throughout pregnancy. Il-6, IL-9, and IL-10 were more commonly detected in WLHIV in the first trimester. Trends of increased detection of Th1 (IL-2, IL-12p70), Th2 (IL-4, Il-5, Il-13) and Th17 (IL-17A, Il-17F, IL-21, IL-22) cytokines were associated with small-for-gestational-age babies. Our findings indicate that maternal HIV/ART is associated with distinct systemic cytokine profiles throughout pregnancy.

## Introduction

HIV/AIDS currently affects an estimated 38 million people worldwide including approximately 1.3 million pregnant women, the majority of whom reside in sub-Saharan Africa^1^. A systematic review and meta-analysis demonstrated that WLHIV not receiving antiretroviral therapy (ART) experience higher rates of adverse pregnancy outcomes, including preterm birth, low birth weight, small-for-gestational-age (SGA), and stillbirth, compared to HIV-negative women^2^. Preterm birth is the leading cause of neonatal and child mortality globally, and is associated with significant short- and long-term morbidities^3^. Intra-uterine growth restriction is associated with stillbirth and SGA, which is also associated with neonatal morbidity and mortality^4^. While the administration of ART during pregnancy is effective at improving maternal outcomes and reducing mother-to-child HIV transmission, it does not reverse HIV-associated adverse pregnancy outcomes^5–10^.

Pregnancy is an immunologically dynamic period involving specific temporal adaptations in immune function and regulation^11^. This includes shifts in the prevailing cytokines which correspond to the biological processes occurring at each stage of pregnancy. For instance, in early pregnancy, implantation and placentation involve inflammatory processes as does labour, whereas the second and third trimester are predominantly anti-inflammatory periods^12–14^. HIV infection is characterised by a decline in CD4 T cells, CD8 T cell expansion and a shift from a Th1 to a Th2 immune response. ART aims to suppress viral replication as well as restore CD4/CD8 ratios and redress the Th1/Th2 balance^15^. However, ART administered during pregnancy may disrupt the regular immune programme of pregnancy^16^. We hypothesised that immunological changes caused by maternal HIV and ART might impact immunological changes occurring during pregnancy, which might in turn affect pregnancy outcomes. To gain insight into these immunological processes, we evaluated systemic pro-inflammatory, anti-viral, Th1, Th2, Th17, anti-inflammatory and pleiotropic cytokines and chemokines at each trimester of pregnancy in WLHIV and HIV-negative women in South Africa to investigate how HIV infection and ART impact cytokine responses during pregnancy.

## Results

### Patient characteristics

Fifty-six WLHIV and 68 HIV-negative women were included in this study. Maternal age was greater in WLHIV (p = 0.034) and there was a difference in the number of years of education (p = 0.038) (Table 1). However, there were no differences in either body mass index, obstetric history, smoking or alcohol intake (Table 1). CD4 counts were available for 21 WLHIV, with a median (IQR) of 498 (397–662) cells/mm^3^ (Table 1). Viral loads were only available in early pregnancy in 9 of the WLHIV, with median (IQR) of 97 (23–7029) copies/ml, and at delivery 15 viral loads were available, 12 of which were undetectable and the highest VL detected was 119 copies/ml. There were no cases of mother-to-child transmission of HIV.

**Table 1:** Patient characteristics.

	HIV+	HIV−	*p*
Number of patients	56	68	
Maternal age (mean [SD])	32 [5.2]	30 [5.3]	0.034
Pre-pregnancy body mass index (mean [SD])	27.7 [4.2]	26.4 [3.9]	0.091
Smoking during pregnancy (n)	5 [9%]	2 [3%]	0.148
Alcohol intake during pregnancy (n)	9 [16%]	5 [7%]	0.159
Number of years of education (median [IQR])	12 [11–12]	12 [12–12]	0.038
Number of previous pregnancies (mean [SD])	2 [1.1]	2 [1.3]	0.864
**History of adverse pregnancy outcome (n)**			
Yes	30 [53.6%]	39 [57%]	0.241
No	24 [43%]	22 [32%]	
Unknown	2 [3.6%]	7 [10%]	
Preterm birth (n)	23 [41%]	25 [37%]	0.712
Term birth (n)	33 [59%]	43 [63%]	
Small-for-gestational-age (n)	19 [34%]	11 [16%]	0.055
CD4 count (Trimester 1) (median cells/mm^3^ [IQR]) (n = 21)	498 [397–662]		
**Antiretroviral therapy initiation (n [%])**			
Preconception	21 [38%]	N/A	
Post-conception	23 [41%]	N/A	
Unknown	12 [21%]	N/A	
**Number of samples (n)**			
Trimester 1	54	68	
Trimester 2	45	54	
Trimester 3	31	37	
Delivery	11	0	
Postnatal	18	0	
**Weeks + days of gestation at sample collection (median [IQR])**			
Trimester 1	11 + 6 [10 + 5 − 13 + 1]	12 + 2 [11 + 1 − 13 + 2]	0.311
Trimester 2	26+0 [24 + 6 − 27 + 2]	26+0 [23 + 3 − 27+0]	0.869
Trimester 3	35+0 [32 + 2 − 36+0]	35 + 2 [33+0 − 37+0]	0.398

Characteristics of pregnant women living with HIV (HIV+) and pregnant HIV-negative women (HIV−) enrolled in prospective pregnancy cohort study in Soweto, South Africa, of whom plasma samples were analysed. Statistical comparisons were made using unpaired t test, Mann–Whitney U test, Chi-square test or Fisher’s exact test, as appropriate. History of adverse pregnancy outcome: at least one occurrence of preterm birth, low birth weight, miscarriage, stillbirth or neonatal death.

### Cytokines across pregnancy

**Figure 1:**
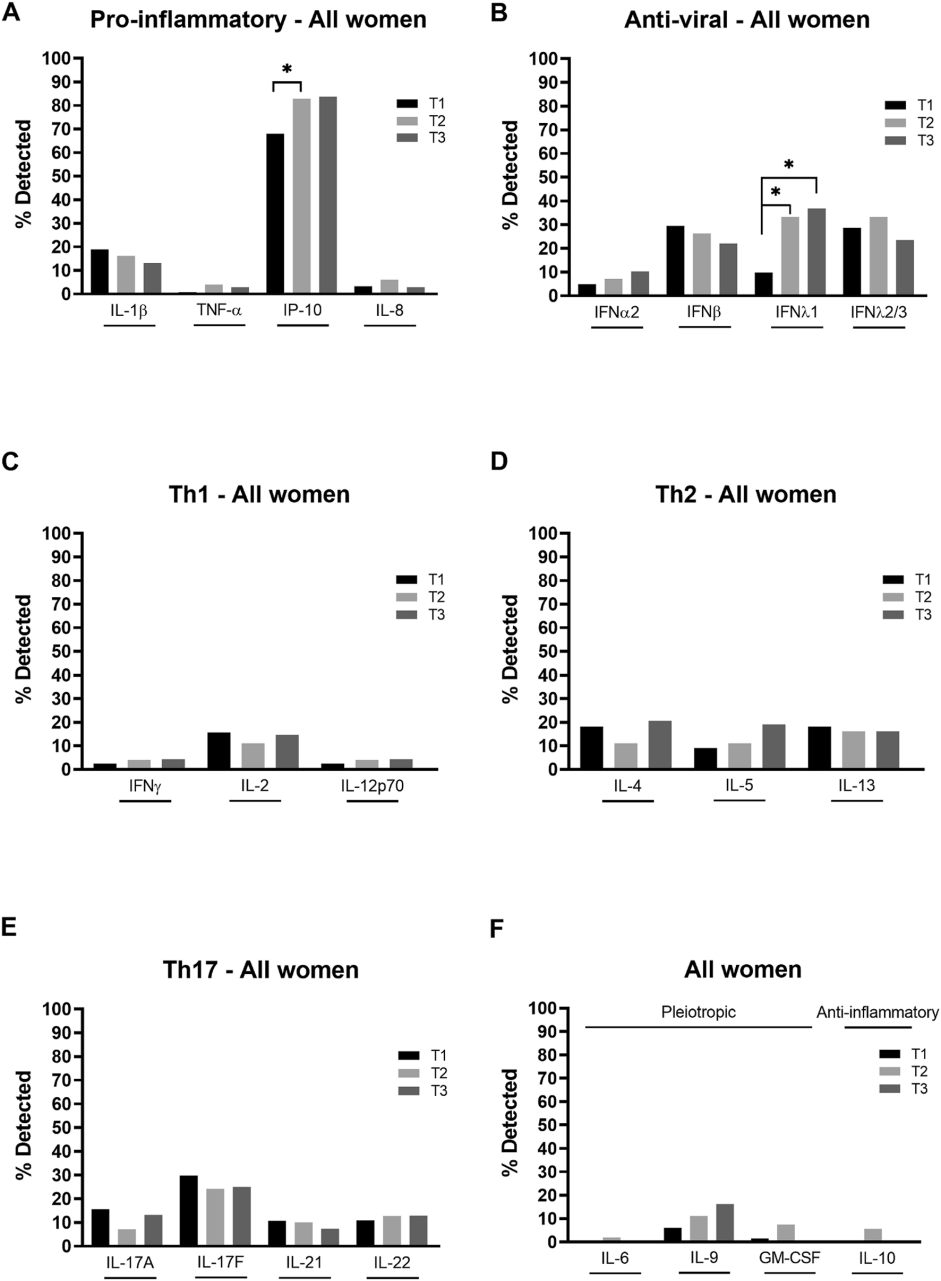
Systemic pro-inflammatory, anti-viral, Th1, Th2, Th17, pleiotropic and anti-inflammatory cytokines across pregnancy. (**A**) Pro-inflammatory (IL-1β, TNF-α, IP-10, IL-8), (**B**) anti-viral (IFNα2, IFNβ, IFNλ1, IFNλ2/3), (**C**) Th1 (IFNγ, IL-2, IL-12p70), (**D**) Th2 (IL-4, IL-5, IL-13), (**E**) Th17 (IL-17A, IL-17F, IL-21, IL-22) and (**F**) pleiotropic and anti-inflammatory (IL-6, IL-9, GM-CSF and IL-10 respectively) cytokines were measured in maternal plasma collected during the first (T1), second (T2) and third (T3) trimester. Data are presented as the percentage of samples in which cytokines were detected by multiplex cytokine assay. *P < 0.05.

IL-1β, TNF-α, IP-10, IL-8 (pro-inflammatory), IFNα2, IFNβ, IFNλ1, IFNλ2/3 (anti-viral), IFNγ, IL-2, IL-12p70 (Th1), IL-4, IL-5, IL-13 (Th2), IL-17A, IL-17F, IL-21, IL-22 (Th17), IL-6, IL-9, GM-CSF (pleiotropic) and IL-10 (anti-inflammatory) cytokines and chemokines (hereafter referred to as cytokines) were measured in maternal plasma collected at each trimester and reported as a percentage of samples in which cytokines were detected (Fig. 1, Supp. Table 1). Across the 22 cytokines analysed among all the women of the study (i.e. both WLHIV and HIV-negative women), the rate of cytokine detection varied considerably and ranged from 1 to 84% with median detection rates of 11%, 11% and 13% across the three trimesters, respectively. IP-10 had the highest detection rates of 68%, 83% and 84% in the first, second and third trimester, respectively (Fig. 1A–F).

Among all the women studied, there were significant changes throughout the course of pregnancy in the detection of IP-10 and IFNλ1, with an increase in the detection of both cytokines form the first to second trimester, as well as from the first to third trimester for IFNλ1 (Fig. 1A,B). While these were the only significant changes, there were also notable trends of an increase in IFNα2, IL-5 and IL-9 and a decrease in IL-1β and IFNβ detection during pregnancy (Fig. 1A,B,D,F).

### Cytokines across pregnancy in WLHIV and HIV-negative women

**Figure 2:**
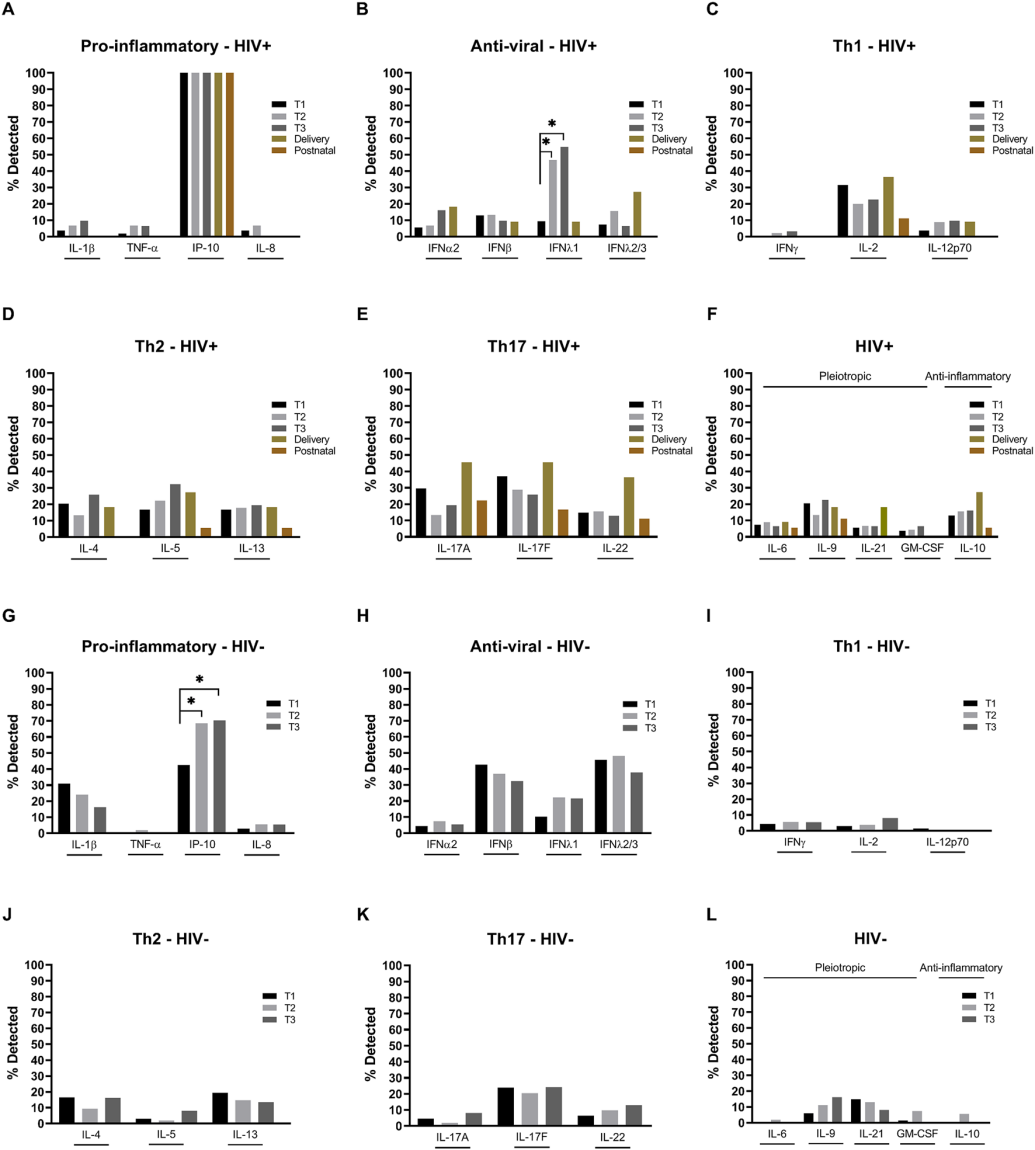
Systemic cytokines across pregnancy in women living with HIV and HIV-negative women. (**A**,**G**) Pro-inflammatory (IL-1β, TNF-α, IP-10, IL-8), (**B**,**H**) anti-viral (IFNα2, IFNβ, IFNλ1, IFNλ2/3), (**C**,**I**) Th1 (IFNγ, IL-2, IL-12p70), (**D**,**J**) Th2 (IL-4, IL-5, IL-13), (**E**,**K**) Th17 (IL-17A, IL-17F, IL-21, IL-22) and (**F**,**L**) pleiotropic and anti-inflammatory (IL-6, IL-9, GM-CSF and IL-10 respectively) cytokines were measured in the maternal plasma collected during the first (T1), second (T2) and third (T3) trimester in women living with HIV (HIV+) (**A**–**F**) and HIV-negative women (HIV−) (**G**–**L**). Data are presented as the percentage of samples in which cytokines were detected by multiplex cytokine assay. *P < 0.05.

Cytokine detection throughout pregnancy was also analysed according to HIV status (Fig. 2, Supp. Table 1). For a number of WLHIV we also had delivery and 6 weeks postnatal samples which were included in the analysis of the WLHIV. Among the pro-inflammatory cytokines, IP-10 was detected in all WLHIV at each time-point and the detection rate of IP-10 increased throughout pregnancy in HIV-negative women (Fig. 2A,G). Therefore, IP-10 was additionally analysed according to cytokine concentration and was found to remain stable across pregnancy in WLHIV; as did IP-10 concentrations across pregnancy in all women (both WLHIV and HIV-negative women) (Supp. Figure 1A,B). There was also a trend for an increase in IL-1β detection over the course of pregnancy in WLHIV, which was in contrast to the decrease in detection seen in the analysis of HIV-negative women (Fig. 2A,G). Detection of IFNλ1, an anti-viral cytokine, increased significantly from the first to the second and third trimesters in WLHIV and there was a trend for an increase and a decrease in IFNα2 and IFNβ detection, respectively, during pregnancy (Fig. 2B). There was also a trend for an increase in the detection of the Th2 cytokine IL-5, and a decrease in the Th17 cytokine IL-17F, over the course of pregnancy in WLHIV (Fig. 2D–F). At delivery in WLHIV there was a trend for increased detection of IFNα2 and IFNλ2/3 of the anti-viral cytokines, IL-2 of the Th1 cytokines, the four Th17 cytokines measured (IL-17A, IL-17F, IL-21 and IL-22), and the anti-inflammatory cytokine IL-10, compared to during pregnancy (Fig. 2B,C,E,F). In contrast, the anti-viral cytokines IFNβ and IFNλ1, the three Th2 cytokines measured (IL-4, IL-5, IL-13), and IL-9, a pleiotropic cytokine, had a trend for decreased detection at delivery compared to during pregnancy in WLHIV (Fig. 2B,D,F). With the exception of IP-10, cytokines that were detected at delivery showed a trend for comparatively lower detection at 6 weeks postnatal in WLHIV (Fig. 2A–F). Many cytokines were not detected in any postnatal samples in WLHIV, including IL-1β, TNF-α, IL-8 (pro-inflammatory), all anti-viral cytokines, IFNγ and IL-12p70 (Th1), IL-4 (Th2), IL-21 (Th17), and GM-CSF (pleiotropic). In contrast, all the cytokines that were detected postnatally in WLHIV (IP-10 (pro-inflammatory), IL-2 (Th1), IL-5, IL-13 (Th2), IL-17A, IL-17F, IL-22 (Th17), and IL-6, IL-9, and IL-10) were also detected in all trimesters and at delivery (Fig. 2A–F).

In HIV-negative women, similar to the analysis of all women, IP-10 detection increased significantly from 43%, to 69% and 70% in the first, second and third trimester, respectively (Fig. 2G, Supp. Table 1) and the IP-10 concentration remained stable across pregnancy (Supp. Figure 1C). The anti-viral cytokine IFNλ1, the Th17 cytokine IL-22 and the pleiotropic cytokine IL-9 also showed a trend for increasing detection over the course of pregnancy (Fig. 2H,K,L). As with the analysis of all women, IL-1β (pro-inflammatory) and IFNβ (anti-viral), showed a trend of decreasing detection over the course of pregnancy in HIV-negative women, as did the Th2 cytokine IL-13 and the Th17 cytokine IL-21 (Fig. 2G,H,J,K). Detection of the Th1 cytokines, particularly IL-2 and IL-12p70, was low in HIV-negative women (Fig. 2I).

### Cytokines in WLHIV versus HIV-negative women

**Figure 3:**
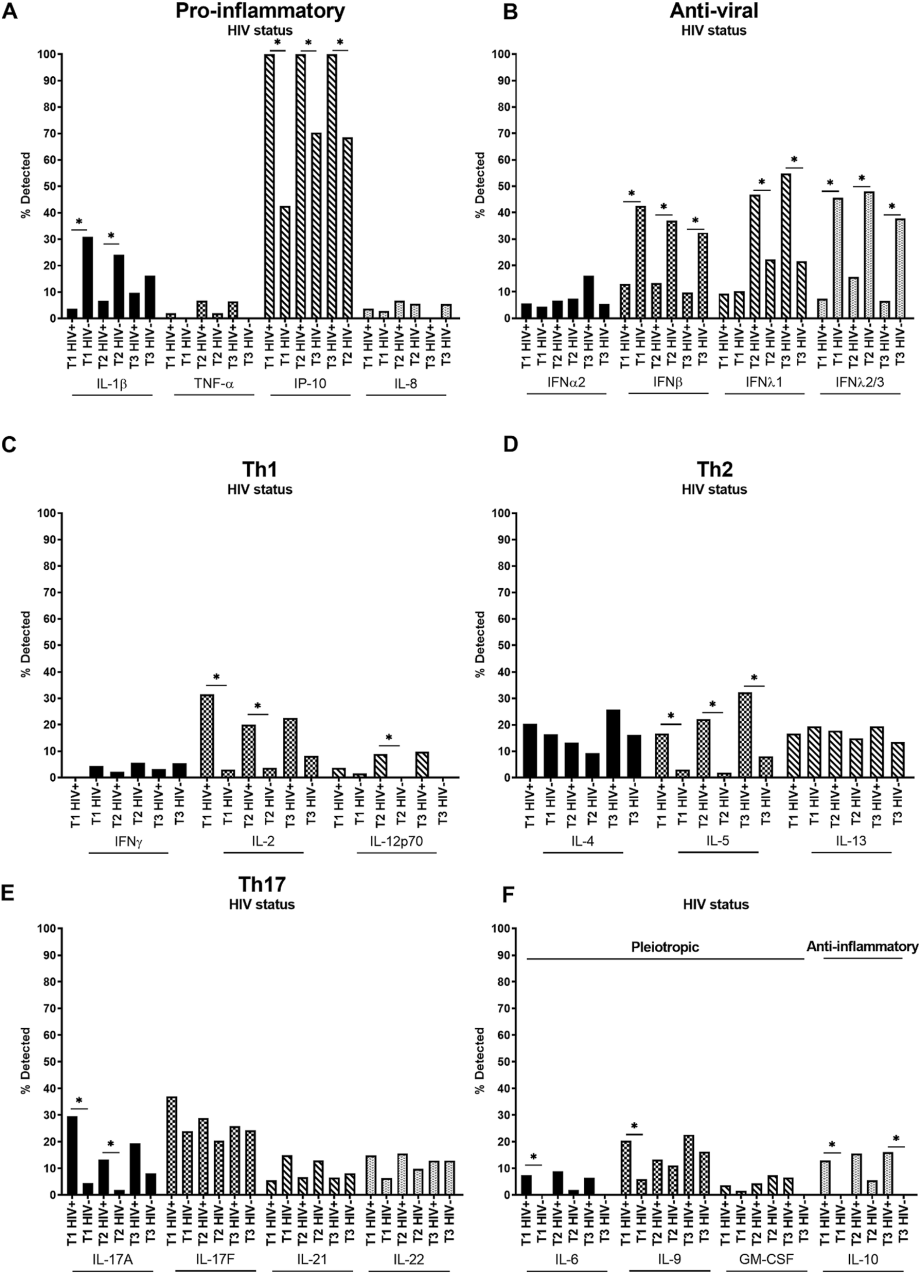
Systemic cytokines in women living with HIV versus HIV-negative women across pregnancy. (**A**) Pro-inflammatory (IL-1β, TNF-α, IP-10, IL-8), (**B**) anti-viral (IFNα2, IFNβ, IFNλ1, IFNλ2/3), (**C**) Th1 (IFNγ, IL-2, IL-12p70), (**D**) Th2 (IL-4, IL-5, IL-13), (**E**) Th17 (IL-17A, IL-17F, IL-21, IL-22) and (**F**) pleiotropic and anti-inflammatory (IL-6, IL-9, GM-CSF and IL-10 respectively) cytokines were measured in maternal plasma collected during the first (T1), second (T2) and third (T3) trimester in women living with HIV (HIV+) and HIV-negative women (HIV−). Data are presented as the percentage of samples in which cytokines were detected by multiplex cytokine assay. *P < 0.05.

As demonstrated in the analysis of WLHIV and HIV-negative women separately, there were differences in the patterns of cytokine detection between the two groups. Therefore, we compared cytokine detection in WLHIV and HIV-negative women over the course of pregnancy (Fig. 3, Supp. Table 2). Of the pro-inflammatory cytokines IL-1β was detected significantly less often in WLHIV compared to HIV-negative women in the first and second trimester, whereas IP-10 was detected in all WLHIV in all three trimesters, significantly more than in HIV-negative women (Fig. 3A). Additionally, IP-10 concentrations were significantly higher in WLHIV compared to HIV-negative women in the first trimester (median 48.50 vs 35.00 pg/ml) and second trimester (median 64.88 vs 26.95 pg/ml) (Supp. Figure 1D). Detection of IFNβ and IFNλ2/3 was significantly lower in WLHIV across all three trimesters, whereas detection of IFNλ1 was significantly greater in WLHIV compared to HIV-negative women in the second and third trimesters (Fig. 3B). There was also a trend for greater third trimester IFNα2 detection in WLHIV compared to HIV-negative women (Fig. 3B). Of the Th1 cytokines, detection of IL-2 was higher in WLHIV in all three trimesters with a significant difference in the first and second trimester; similarly, IL-12p70 detection was greater in WLHIV and significantly so in the second trimester, compared to HIV-negative women (Fig. 3C). In contrast, there was a trend for decreased detection of IFNγ in all three trimesters in WLHIV versus HIV-negative women (Fig. 3C). Detection of the Th2 cytokines IL-4 and IL-5 was greater in WLHIV in all three trimesters, however, the differences were only significant for IL-5 (Fig. 3D). Detection of the Th17 cytokine IL-17A was significantly greater in WLHIV in the first and second trimester with a trend for increased detection in the third trimester compared to HIV-negative women (Fig. 3E). IL-17F and IL-22 of the Th17 cytokines also showed a trend for increased detection, particularly in the first and second trimester, whereas there was a trend for lower detection of IL-21 in WLHIV versus HIV-negative women (Fig. 3E). IL-6 and IL-9, both pleiotropic cytokines, were detected at higher rates in WLHIV compared to HIV-negative women across all trimesters with a significant difference in the first trimester (Fig. 3F). Similarly, detection of the anti-inflammatory cytokine IL-10 was greater in WLHIV across all trimesters and significantly greater in the first and third trimester compared to HIV-negative women, in which IL-10 was not detected in either trimester (Fig. 3F).

### Cytokines and adverse pregnancy outcomes

Maternal HIV infection and ART have been associated with an increased risk of adverse perinatal outcomes, including preterm birth and small-for-gestational-age infants^2,9^. Peripheral frequencies of a number of immune cells, including CD4 + T cells, γδ T cells, MAIT cells, and innate lymphoid cells (ILCs) have been associated with HIV infection as well as adverse perinatal outcomes, in particular preterm birth^17–19^. Therefore, we compared cytokine detection between women who delivered preterm versus those who delivered at term, as well as between women who delivered small-for-gestational-age (SGA) infants versus those who did not.

**Figure 4:**
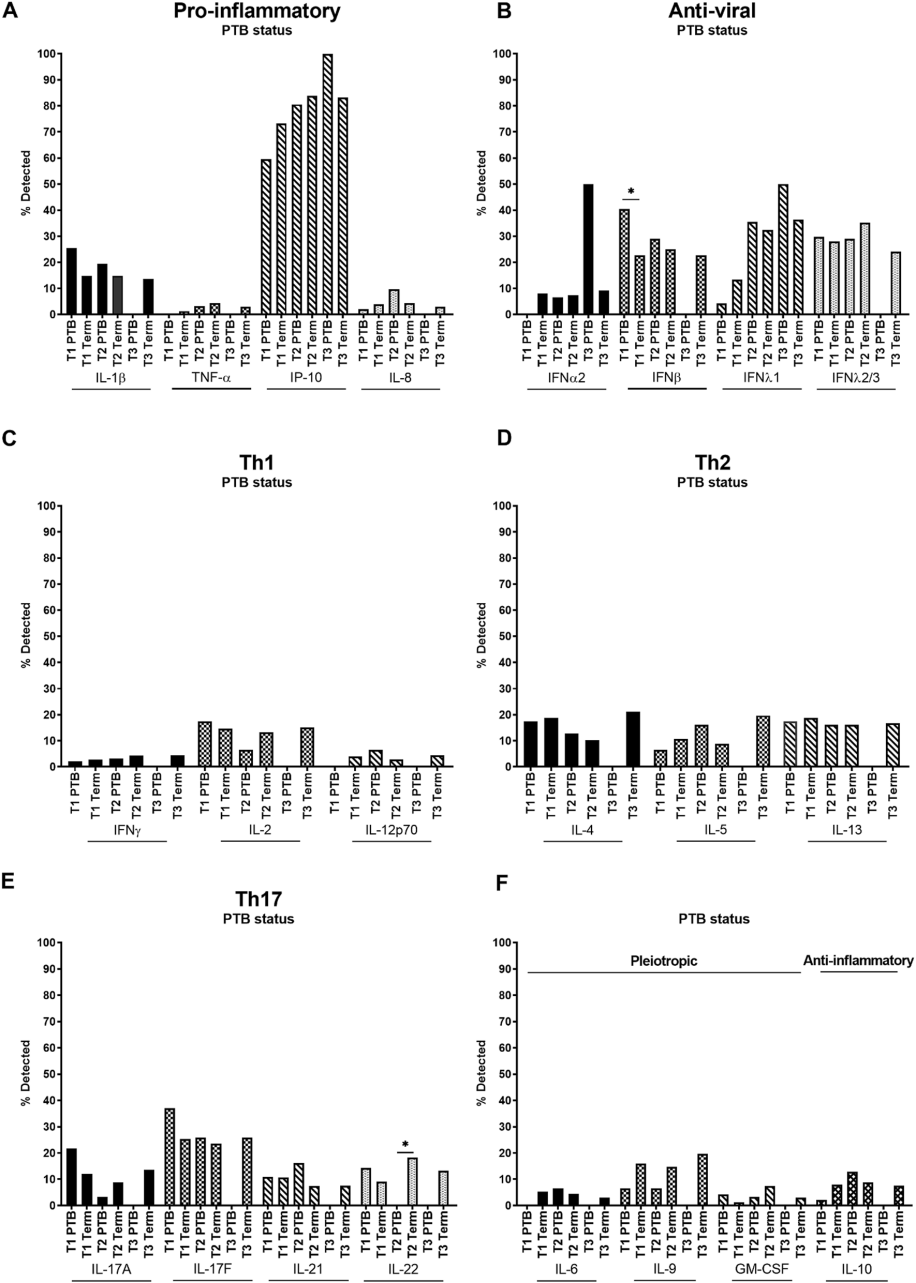
Systemic cytokines across pregnancy in women who went on to deliver preterm (PTB) versus women who delivered at term. (**A**) Pro-inflammatory (IL-1β, TNF-α, IP-10, IL-8), (**B**) anti-viral (IFNα2, IFNβ, IFNλ1, IFNλ2/3), (**C**) Th1 (IFNγ, IL-2, IL-12p70), (**D**) Th2 (IL-4, IL-5, IL-13), (**E**) Th17 (IL-17A, IL-17F, IL-21, IL-22) and (**F**) pleiotropic and anti-inflammatory (IL-6, IL-9, GM-CSF and IL-10 respectively) cytokines were measured in maternal plasma collected during the first (T1), second (T2) and third (T3) trimester. Data are presented as the percentage of samples in which cytokines were detected by multiplex cytokine assay. *P < 0.05.

Although there were no significant differences among pro-inflammatory cytokines, IL-1β showed a trend of increased detection in the first and second trimester in women who would go on to deliver preterm, whereas detection of TNF-α and IP-10 tended to be lower in women who went on to deliver preterm versus term (Fig. 4A). First trimester detection of IFNβ, an anti-viral cytokine, was significantly greater in women who delivered preterm versus those who delivered at term (Fig. 4B). The Th1 cytokine IL-2 and the Th17 cytokines IL-17A, IL-17F and IL-22 tended to decrease from the first to second trimester in women who went on to deliver preterm but tended to remain stable in women who delivered at term (Fig. 4C,E). In contrast, the Th2 cytokine IL-5, tended to increase from the first to second trimester in women who went on to deliver preterm compared to women who delivered at term (Fig. 4D). The Th17 cytokine IL-22 was not detected in the second trimester in any women who went on to deliver preterm, a significant reduction compared to women who delivered at term (Fig. 4E). It should be noted that there were few third trimester PTB samples due to delivery before planned sample date (Fig. 4A–F).

**Figure 5:**
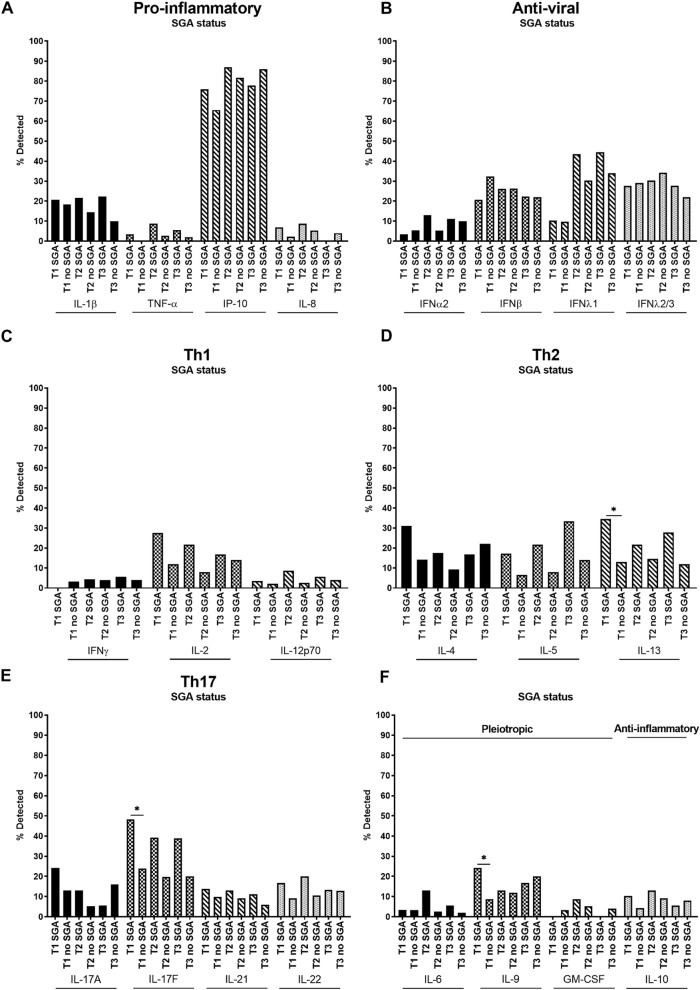
Systemic cytokines across pregnancy in women who went on to deliver small-for-gestational-age (SGA) infants versus women who delivered non-SGA infants. (**A**) Pro-inflammatory (IL-1β, TNF-α, IP-10, IL-8), (**B**) anti-viral (IFNα2, IFNβ, IFNλ1, IFNλ2/3), (**C**) Th1 (IFNγ, IL-2, IL-12p70), (**D**) Th2 (IL-4, IL-5, IL-13), (**E**) Th17 (IL-17A, IL-17F, IL-21, IL-22) and (**F**) pleiotropic and anti-inflammatory (IL-6, IL-9, GM-CSF and IL-10 respectively) cytokines were measured in maternal plasma collected during the first (T1), second (T2) and third (T3) trimester. Data are presented as the percentage of samples in which cytokines were detected by multiplex cytokine assay. *P < 0.05.

In the analysis of women with and without SGA babies, among pro-inflammatory cytokines there was a trend towards higher detection of all cytokines in SGA compared to non-SGA pregnancies, in particular in the first and second trimesters (Fig. 5A). Among anti-viral cytokines, IFNα2 and IFNλ1 also showed higher detection in SGA cases compared to non-SGA cases (Fig. 5B). Among Th1 cytokines, IL-2 and IL-12p70 were higher in SGA compared to non-SGA cases in all trimesters, although these trends were not statistically significant (Fig. 5C). All Th2 cytokines, IL-4, IL-5, and IL-13, showed trends of increased detection across pregnancy in women who delivered SGA infants (Fig. 5D). IL-13 detection was significantly greater in the first trimester in women who went on to deliver SGA infants (Fig. 5D). Additionally, all Th17 cytokines, IL-17A, IL-17F, IL-21 and IL-22, showed trends of increased detection in women who went on to deliver SGA infants versus those who did not, across pregnancy (Fig. 5E). Detection of the IL-17F was significantly greater in the first trimester in women who delivered SGA infants (Fig. 5E). Increased detection of the pleiotropic cytokine IL-9 in the first trimester was significant in women who went on to deliver SGA infants compared those who did not (Fig. 5F).

### Cytokine subgroups

**Figure 6:**
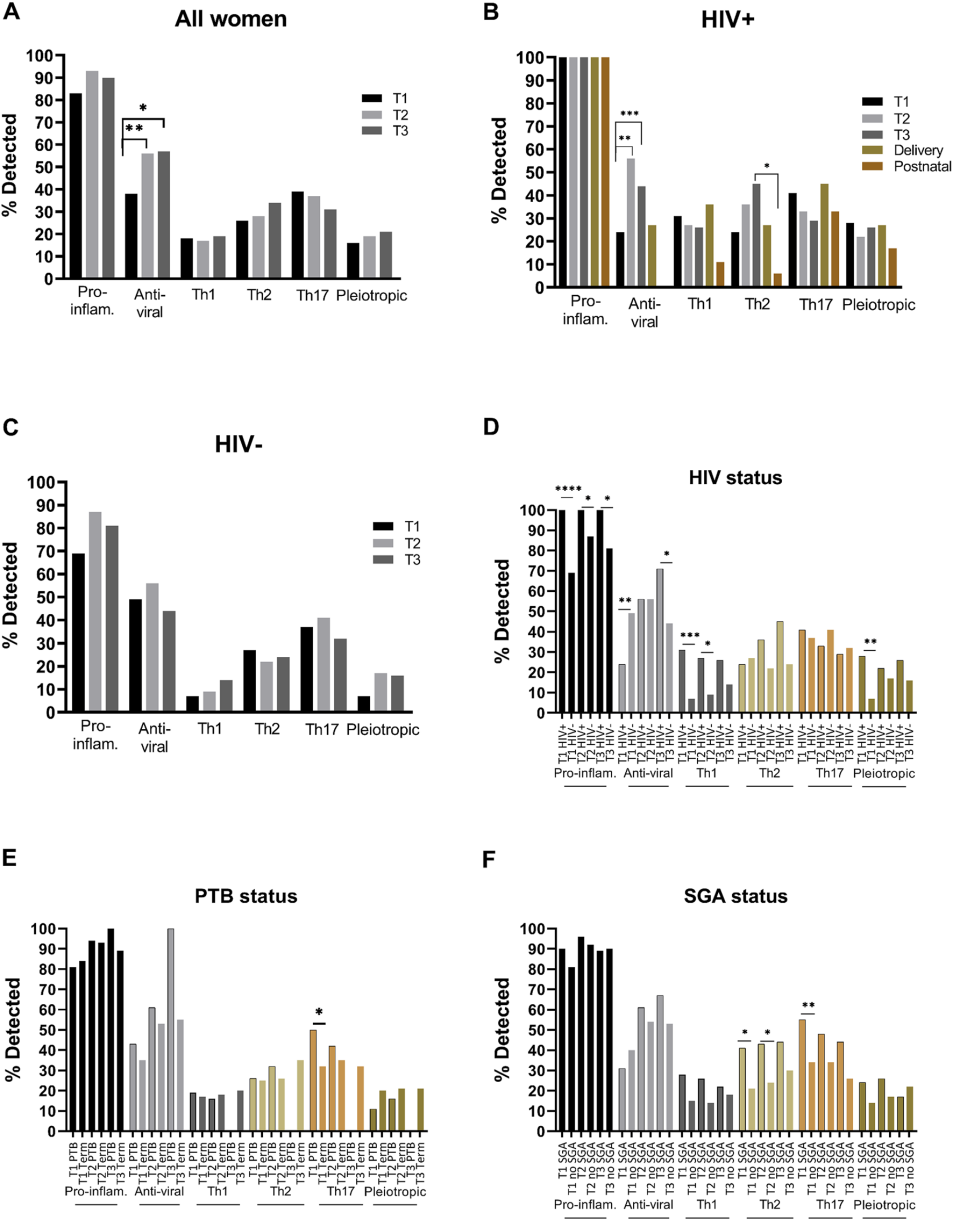
Systemic cytokine subgroups across pregnancy. (**A**) All women, (**B**) women living with HIV (HIV+), (**C**) HIV-negative women (HIV-), (**D**) HIV+ and HIV− women, (**E**) preterm (PTB) and term birth, and (**F**) small-for-gestational-age (SGA) and non-SGA infants. Pro-inflammatory (IL-1β, TNF-α, IP-10, IL-8), anti-viral (IFNα2, IFNβ, IFNλ1, IFNλ2/3), Th1 (IFNγ, IL-2, IL-12p70), Th2 (IL-4, IL-5, IL-13), Th17 (IL-17A, IL-17F, IL-21, IL-22) and pleiotropic (IL-6, IL-9, GM-CSF and IL-10 respectively) cytokines were measured in maternal plasma collected during the first (T1), second (T2) and third (T3) trimester in women living with HIV (HIV+) and HIV-negative (HIV−) women. Data are presented as the percentage of samples in which subgroups of cytokines were detected by multiplex cytokine assay. *P < 0.05, **P < 0.01, ***P < 0.001, ****P < 0.0001.

In an effort to summarise the data on the broad range of cytokines, we aggregated the data according to detection of different cytokine subgroups, i.e. pro-inflammatory, anti-viral, Th1, Th2, Th3, Th17 and pleiotropic cytokines (Fig. 6, Supp. Tables 3, 4). Among all women, we found an increase in detection of anti-viral cytokines from the first to the second trimester, as well as from the first to the third trimester (Fig. 6A). Among WLHIV, a similar increase in detection of anti-viral cytokines from the first to the second and third trimesters was seen. In addition, among WLHIV a significant drop in detection of Th2 cytokines was seen from the third trimester to 6 weeks postnatally (Fig. 6B). Among HIV-negative women no significant changes were seen during pregnancy (Fig. 6C). In the comparison of WLHIV and HIV-negative women, significant differences were seen in the detection of pro-inflammatory, antiviral, Th1 and pleiotropic cytokines (Fig. 6D). Pro-inflammatory cytokines were detected more often among WLHIV than HIV-negative women in each trimester. Anti-viral cytokines were detected more often among HIV-negative women in the first trimester, but more often among WLHIV in the third trimester. Th1 cytokines were detected more often among WLHIV in each trimester, and significantly so in the first and second trimesters. Pleiotropic cytokines were detected more often among WLHIV in the first trimester (Fig. 6D). Th17 cytokines were detected more often in the first trimester of women who would go on to deliver preterm compared to women who delivered at term (Fig. 6E). Th2 cytokines were detected more often in each trimester among women with SGA newborns, and significantly so in the first and second trimesters. In addition, Th17 cytokines were detected more often among women with SGA newborns, which reached statistical significance in the first trimester (Fig. 6F).

## Discussion

In this study we evaluated a broad range of cytokines at each trimester of pregnancy in WLHIV and HIV-negative women in South Africa. We found that the pro-inflammatory cytokine IP-10 was detected in each trimester in all WLHIV, which was significantly more than in HIV-negative women. The anti-viral cytokine IFNλ1 was detected more frequently in WLHIV, whereas IFNβ and IFNλ2/3 were detected more frequently in HIV-negative women. Th1 cytokines IL-12 and IL-12p70, Th2 cytokine IL-5, and Th17 cytokine IL-17A were detected more frequently in WLHIV throughout pregnancy. Il-6, IL-9, and IL-10 were more commonly detected in WLHIV in the 1^st^ trimester. Trends of increased detection of Th1 (IL-2, IL-12p70), Th2 (IL-4, Il-5, Il-13) and Th17 (IL-17A, Il-17F, IL-21, IL-22) cytokines were also associated with small-for-gestational-age babies. Analysis of cytokine subgroups showed that pro-inflammatory, Th1 and pleiotropic cytokines were more often detected among WLHIV, compared to HIV-negative women. Preterm birth was associated with increased detection of Th17 cytokines, and SGA was associated with increased detection of Th2 and Th17 cytokines.

IP-10 is an inflammatory chemokine involved in the migration and adhesion of immune cells including CD4 + and CD8 + T cells and NK and NKT cells during an inflammatory response^20^. This chemokine is secreted by a range of cell types, including monocytes, leukocytes, neutrophils and endothelial cells, predominantly in response to IFNγ^20,21^. The combined analysis of all women revealed significant increases in the detection of IP-10 over the course of pregnancy. This was attributable to HIV-negative women, in whom detection of IP-10 increased significantly from the first to the second and third trimester. Similar to our findings, peripheral IP-10 levels during pregnancy in HIV-negative women have been shown to increase over time^22,23^. However, our analysis of IP-10 concentration did not show a significant increase in IP-10 over pregnancy in the women analysed. HIV infection is associated with elevated peripheral IP-10 levels which are indicative of HIV disease progression and positively associated with HIV viral load^24^. While IP-10 levels in people living with HIV (PLHIV) are lowered by ART, they remain higher than controls^24^. This was demonstrated in our analysis, in which IP-10 was detected in all WLHIV at all three trimesters, as well as at delivery and 6 weeks postnatal, and therefore had significantly greater detection rates in WLHIV compared to HIV-negative women. Labour is associated with an infiltration of inflammatory cells and an increase in inflammatory cytokines, including IP-10, IL-1β, TNF-α and IL-8, in tissues including the cervix, myometrium and chorio-amniotic membranes^13,25–28^. Therefore, the observed increase in IP-10 detection during the course of pregnancy in HIV-negative women may align with an increase in inflammatory processes towards the end of pregnancy involved in labour. In the decidua IP-10 levels are correlated with decidual macrophage numbers and IP-10 is expressed by monocytes and myeloid dendritic cells in the periphery in PLHIV^27,29^. Elevated peripheral and decidual IP-10 levels have been associated with preeclampsia^30,31^ and suggests WLHIV may be at increased risk of preeclampsia^32^. In our analysis of women who experienced preterm versus term births and women with and without small-for-gestational-age infants, there were no significant differences in IP-10 detection. This is in accordance with a study which found no difference in peripheral IP-10 levels between women with and without small-for-gestational-age babies^30^, suggesting IP-10 is involved in specific rather than generalised pathologies of pregnancy.

The remaining pro-inflammatory cytokines, IL-1β, TNF-α and IL-8, did not show any statistically significant changes in detection over the course of pregnancy. However, there was a striking opposite trend in the detection of IL-1β throughout pregnancy in WLHIV and HIV-negative women, with an upward trend from the first to third trimester in WLHIV and the opposite in HIV-negative women. Additionally, this was associated with significantly lower IL-1β detection rates in WLHIV compared to HIV-negative women. Studies in HIV-negative women have shown that peripheral IL-1β levels decrease over pregnancy, including in plasma^33,34^, suggesting increases in IL-1β over the course of pregnancy are specific to HIV infection. However, other studies suggest IL-1β levels are not changed over pregnancy or have a positive association with gestational weeks^22,23^. Elevated levels of peripheral IL-1β, TNF-α and IL-8, as well as IL-6, are associated with pre-eclampsia in pregnancy and are raised during HIV infection^35–37^. While we did not find significant differences in the detection rates of these cytokines according to preterm birth or small-for-gestational age status, IL-1β detection tended to be greater in women who delivered preterm and small-for-gestational-age infants.

Th1 cytokines generally mediate cellular immune responses against intracellular bacteria and viruses, while Th2 cytokines are associated with antibody responses and protection against extracellular parasites. Pregnancy is described as a Th2 predominated condition and shifts in Th1/Th2 cytokine ratios have been associated with conditions such as preeclampsia and preterm birth^16,32,38^. HIV infection is also classified as a Th2 biased condition in which ART redresses the Th1/Th2 balance in PLHIV and therefore may have detrimental consequences for pregnancy^16^. In our data set Th1 cytokine detection was particularly low in HIV-negative women and there were significantly higher detection rates of IL-2 and IL-12p70 in WLHIV. In addition, of the Th2 cytokines, IL-4 detection tended to be greater and IL-5 detection significantly greater in WLHIV. IL-13 remained stable across pregnancy and between WLHIV and HIV-negative women, as well as between women who delivered preterm and at term. In contrast, IL-13 detection was greater in women who delivered small-for-gestational-age infants, particularly in the first trimester, and IL-4 and IL-5 detection also tended to be greater in this group, as well as IL-2 and IL-12p70 of the Th1 cytokines. Overall, this data suggests that HIV/ART modulates both Th1 and Th2 cytokine responses during pregnancy. While HIV/ART mediated Th1/Th2 cytokine shifts have been associated with pathological pregnancy conditions, the immune responses associated with both HIV/ART and pregnancy are complex, and the increases in both Th1 and Th2 cytokines associated with HIV/ART suggest that it may not involve a clear-cut shift from one inflammatory state to another.

Three Th17 cytokines, IL-17A, IL-17F and IL-22, tended to be detected more often in WLHIV, with significantly greater detection of IL-17A in the first and second trimester compared to HIV-negative women. IL-17A is a pro-inflammatory cytokine produced by CD4 + Th17 helper cells, which also secrete TNFα, IL-1, IL-2, IL-21 and IL-22. Both Th17 cells and IL-17 are found in increased proportions in the peripheral blood and decidua of women who experience preterm birth, miscarriage and preeclampsia^39–41^. In our data IL-17A detection tended to drop in preterm birth mothers from the first to the second trimester, potentially demonstrating increased recruitment to the decidua towards the end of pregnancy. Similar to IL-17A, IL-17F is a pro-inflammatory cytokine produced by Th17 cells and in our study tended to be detected more often in WLHIV. In addition, IL-17F was more commonly detected in women who went on to have small-for-gestational-age infants versus those who did not, particularly in the first trimester (48% versus 24%). Th17 cells are preferentially lost from the gastrointestinal tract of HIV-infected individuals, which can be reversed by HAART. Furthermore, other cells, such as Vδ1 T cells, can make IL-17 in vivo during HIV infection and may contribute to the greater detection of IL-17 in WLHIV in our study^42^. Th17 cells have been reported to be both depleted, unchanged and increased during HIV infection^42–45^ but our findings of increased peripheral IL-17 detection in pregnancy may suggest WLHIV are at increased risk of IL-17 induced pregnancy complications. Viremia is known to be associated with changes in inflammatory markers, including IL-17^15,43,46^. Unfortunately we had few data on the viral loads of the WLHIV in our study. However, where available the viral loads were generally low. In addition, data from the prospective pregnancy cohort as a whole showed that the vast majority of women received ART and there were no cases of HIV transmission to newborns, indicating good viral suppression in the cohort overall^10^. IL-22 has been shown in mouse models of LPS-induced preterm birth to be important in protection against inflammation-induced preterm birth and IL-22 expression is lower in the decidua of women who experience recurrent pregnancy loss^47,48^. Our finding of significantly lower detection of peripheral IL-22 in women with preterm birth, may be reflective of a decline in recruitment of IL-22 producing cells, e.g. Th17 cells, to the decidua of women with preterm birth.

Detection of the anti-inflammatory cytokines IL-10 and the pleiotropic cytokines IL-6 and IL-9 was also higher in WLHIV. IL-6 is deemed a specific indicator of infection-associated preterm labour and peripheral increases, as well as at the maternal–fetal interface and within the amniotic and cervicovaginal fluid is deemed a risk factor for preterm birth^49–52^. However, while we saw higher IL-6 detection in WLHIV compared to HIV-negative women, in our data there were no differences in IL-6 between women who delivered preterm and at term. This could be due to several factors such as differences in sampling time-points, sample size and the presence or absence of infection. During pregnancy peripheral IL-9 levels positively associate with pregnancy progression^22^ and in our data this was demonstrated in HIV-negative women in whom IL-9 detection tended to increase over the course of pregnancy. Similarly, in women who did not go on to deliver small-for-gestational-age infants, IL-9 detection tended to increase from the first to third trimester. However, IL-9 detection tended to be lowest in the second trimester in WLHIV and in women who went on to deliver small-for-gestational-age infants. In a mouse model of pregnancy uterine levels of IL-9 have been shown to increase during pregnancy and be the most abundant cytokine measured in the uterus and uterine leukocytes^53^. Produced by a range of cell types including Th9 cells, Th2 cells, Th17 cells, Treg cells, mast cells and NK cells, IL-9 is involved in immune tolerance as well as inflammatory disorders^54^ and suggests IL-9 mediates a balance between the anti- and pro-inflammatory responses required for successful uncomplicated pregnancy.

In the analysis of all women, detection of anti-viral cytokine IFNλ1 significantly increased over the course of pregnancy and was attributed to significant increases in detection of the cytokine in WLHIV. In all women, IFNβ detection tended to decrease during pregnancy and IFNλ2/3 detection tended to peak during the second trimester. Type I (IFNα/β) and type III (IFNλs) IFNs are antiviral cytokines which initiate antiviral immune responses against a number of viruses^55,56^. In pregnancy, type III IFNs are constitutively released from human trophoblast cells and are involved in protection of the placenta against viral infection, including against HIV; and the potential contribution of maternal peripheral IFNλ may explain the significant increases in IFNλ1 detection we see in WLHIV^57,58^. In contrast, detection of both IFNβ and IFNλ2/3 was significantly lower in WLHIV at all three trimesters compared to HIV-negative women. In another study investigating systemic cytokine levels during acute HIV infection, IFNβ levels were not elevated^29^ suggesting the lower IFN detection rates maybe a result of HIV infection that is maintained during pregnancy. For instance, HIV infection is associated with chronic immune activation which, as well as the activation of immune pathways, also involves immune exhaustion, including a diminished capacity of plasmacytoid DCs, the main type I IFN producer, to secrete type I IFN^59^. In mice, type I IFNs are expressed in tissues including the decidua suggesting a role in pregnancy, however, type I IFN signalling has also been suggested to prime for inflammation driven preterm birth^60,61^. In support of this, we find that IFNβ detection is significantly increased in preterm birth mothers.

Our study has some strengths and limitations. Strengths of our study included the prospective study design, with samples collected in each trimester of pregnancy. Pregnancy outcomes were accurately determined based on a first trimester dating ultrasound and standardised birthweight measurement within 24 h of birth. Furthermore, we assayed a broad range of cytokines in parallel to obtain a comprehensive view of systemic cytokine levels. Limitations of the study included the limited information regarding viral loads and CD4 counts, which limited interpretation of our results to some extent. The sources of the cytokines, in terms of cell types and their location, remain unknown and as a consequence it is uncertain how differences and changes in systemic cytokine detection correlate with changes in the uterus.

Successful pregnancy involves the coordination of multiple immune processes and redundancy in immune function may suggest adverse outcomes result from the dysregulation of multiple processes^11,62^. Indeed, cytokines exemplify this complexity by their overlapping and redundant functions. We found significant differences in detection rates of individual cytokines between WLHIV and HIV-negative women throughout pregnancy, as well as differences associated with adverse pregnancy outcomes. Our findings therefore indicate that maternal HIV/ART is associated with distinct systemic cytokine profiles throughout pregnancy and further work is needed to elucidate the mechanisms that underlie the increased risk of adverse pregnancy outcomes.

## Methods

### Patient population and sample collection

Between 27 November 2013 and 20 October 2015, plasma samples were obtained from women enrolled in a prospective pregnancy cohort study at Chris Hani Baragwanath Academic Hospital (CHBAH), Soweto, South Africa^10^. Women included in the study were black South African, living in Soweto, aged 18 years or over, with a spontaneous conception resulting in a singleton pregnancy. Women with multiple pregnancies, a body mass index > 35 kg/m^2^ or an intellectual or physical disability, were excluded. All women had a first trimester dating ultrasound scan and HIV testing was routinely offered to those not known to be HIV-positive at enrolment. All WLHIV received antenatal antiretroviral therapy, mainly efavirenz-based HAART^10^. Data on timing of ART initiation was also collected. ART initiation was defined as preconception if started before the date of the last menstrual period or post-conception if initiated after the last menstrual period date. Among women initiating ART post-conception, this occurred before the first trimester sample date in the majority of women (14 out of 23), with the remaining WLHIV initiating ART after the first trimester sample. Medical and obstetric history were collected from medical records, antenatal cards and/or interviews, and perinatal outcomes of interest were recorded at delivery, including standardised birthweight measurement within 24 h of birth, as previously reported^10^. Preterm birth (PTB) was defined as birth from 16^+0^ to 36^+6^ weeks gestation and infants small-for-gestational age (SGA) defined as newborns under the 10th centile of the INTERGROWTH‐21st Newborn Standard birth‐weight‐for‐gestational‐age/sex^63^. Trained study nurses collected peripheral blood samples from WLHIV and HIV-negative pregnant women during the first, second and third trimester, as previously described^17–19^. Additional samples were collected at delivery and 6 weeks postnatally from a subset of the WLHIV. Samples were separated into plasma and peripheral blood mononuclear cells by standard density gradient centrifugation. Plasma samples were initially stored at − 80 °C. Samples were shipped to Oxford on dry ice where they were stored in liquid nitrogen.

### Cytokine analysis

The Biolegend LEGENDplex™ bead-based multiplex immunoassays were performed using the human anti-virus response (740390) and human Th cytokine (740759) panels to measure IL-1β, IL-6, TNF-α, IP-10, IFNλ1 (IL-29), IL-8, IL-12p70, IFNα2, IFNλ2/3 (IL-28A/B), GM-CSF, IFNβ, IL-10, IFNγ, and IL-5, IL-13, IL-2, IL-9, IL-17A, IL-17F, IL-4, IL-21 and IL-22, respectively, in plasma. The kits were used according to the manufacturer’s instructions. At the end of the protocol samples were sterilised by incubation with 4% paraformaldehyde in 1X PBS then washed and resuspended in the 1X Wash Buffer supplied with the kit. All samples were tested in duplicate. The average MFI of duplicates was used to determine the final cytokine concentration of each sample. Samples testing below the sensitivity of the assay were categorised as not detected. None of the samples tested above the sensitivity of the assay. Samples were read on a LSR II flow cytometer [Becton Dickinson] and analysed using the LEGENDplex™ Data Analysis V8.0 software.

### Statistical analysis

Patient characteristics were compared using the unpaired t test or Mann–Whitney U test for continuous variables and Chi-squared test or Fisher’s exact test for categorical variables. Cytokine detection rates and concentrations ranged widely and for many cytokines the detection rates were low (i.e. few patient samples had a detectable level of the respective cytokines). Reporting cytokine concentrations of all samples would have required making assumptions about the concentrations present in those samples which were below the detection limit. Therefore, we report instead cytokine detection rates (i.e. the percentages of samples in which cytokines are detected) in order to utilise all the data collected without substituting arbitrary values and introducing bias. For those cytokines detected in the majority of samples (i.e. IP-10) we also report analysis of the concentrations. Cytokine detection was also aggregated for the different cytokine subgroups: pro-inflammatory, anti-viral, Th1, Th2, Th17 and pleiotropic cytokines. In this case, for each patient at each time point the detection of cytokines in each subgroup was assessed and if one or more of the cytokines in a subgroup were detected (e.g. IL-1β), then the subgroup (e.g. pro-inflammatory cytokines) was considered to be detected.

The association of cytokine detection with trimester, HIV status, preterm birth status or small-for-gestational age status was analysed using the Chi-Squared or Fisher's exact test as appropriate. Corrected pairwise comparisons were calculated for the association between cytokine detection and time periods, to determine specific pairwise differences between two periods (trimesters one, two, three, delivery or postnatal). The Bonferroni method was used to correct for multiple comparisons. P values < 0.05 were considered statistically significant and analyses were performed using SPSS^®^ version 26.

### Ethical approval

Written informed consent was obtained from all study participants upon enrolment. Ethical approval was obtained from the University of Oxford Tropical Research Ethics Committee (OxTREC) and the Human Research Ethics Committee (Medical) of the University of Witwatersrand, Johannesburg, South Africa. All experiments were performed in accordance with relevant guidelines and regulations.

## Supplementary Information

Supplementary Information 1.
