# Supplementary material for: Maternal HIV infection is associated with distinct systemic cytokine profiles throughout pregnancy in South African women
Source: Sci Rep. 2021 May 12;11:10079. doi: 10.1038/s41598-021-89551-3 (PMC8115111; doi:10.1038/s41598-021-89551-3)
Supplement: Supplementary file 1 — Supplementary Information 1. [file 41598_2021_89551_MOESM1_ESM.pdf]

Supplementary data

Title

Maternal HIV infection is associated with distinct systemic cytokine profiles throughout pregnancy in South African women

Authors

Charlene Akoto[1], Shane A. Norris[2], Joris Hemelaar[2,3]*

Affiliations

[1] Nuffield Department of Women's & Reproductive Health, University of Oxford, The Women's Centre, John Radcliffe Hospital, Oxford, United Kingdom.

[2] South African Medical Research Council Developmental Pathways for Health Research Unit, Department of Paediatrics, School of Clinical Medicine, University of the Witwatersrand, Johannesburg, South Africa.

[3] National Perinatal Epidemiology Unit, Nuffield Department of Population Health, University of Oxford, Oxford, United Kingdom.

*** Correspondence:**

Dr Joris Hemelaar

National Perinatal Epidemiology Unit, Nuffield Department of Population Health,

University of Oxford, Richard Doll Building, Old Road Campus, Oxford, OX3 7LF,

United Kingdom.

Email: jorishemelaar@gmail.com

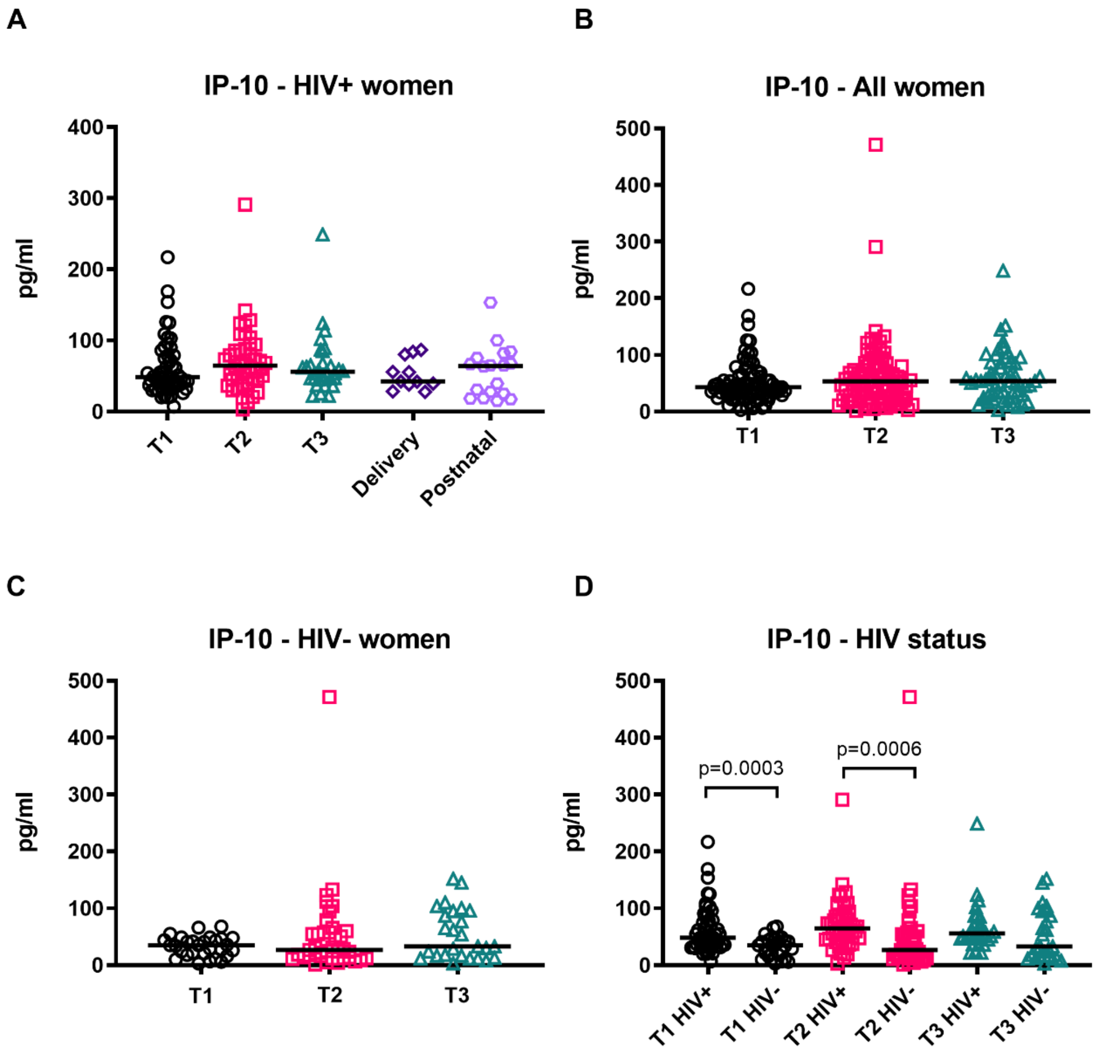

Supplementary Figure 1. Systemic IP-10 concentrations across pregnancy. IP-10 concentration was measured in maternal plasma collected during the first (T1), second (T2) and third (T3) trimester in women living with HIV (HIV+ women) and HIV-negative women (HIV- women), as well as at delivery and six weeks postnatal for n=11 and n=18 HIV+ women, respectively. IP-10 measured in **(A)** HIV+ women, **(B)** all women (both HIV positive and HIV negative), **(C)** HIV- women and **(D)** HIV+ versus HIV- women. The Mann-Whitney U-test was used to compare median values between two groups within a trimester. The Kruskal-Wallis test was used to compare median values of more than two groups followed by Dunn's multiple comparisons test to determine differences between two groups.

	Trimester										
	1		2		3		Delivery		Postnatal		*P*
	n		n		n		n		n		
**All Women**											
**IL-1β**	23_{a}	18.9%	16_{a}	16.2%	9_{a}	13.2%					0.625
**TNF-α**	1_{a}	0.8%	4_{a}	4.0%	2_{a}	2.9%					0.261
**IP-10**	83_{a}	68.0%	82_{b}	82.8%	$57_{a,b}$	83.8%					**0.01**
**IL-8**	4_{a}	3.3%	6_{a}	6.1%	2_{a}	2.9%					0.559
**IFNα2**	6_{a}	4.9%	7_{a}	7.1%	7_{a}	10.3%					0.374
**IFNβ**	36_{a}	29.5%	26_{a}	26.3%	15_{a}	22.1%					0.528
**IFNλ1**	12_{a}	9.8%	33_{b}	33.3%	25_{b}	36.8%					**0.00**
**IFNλ2/3**	35_{a}	28.7%	33_{a}	33.3%	16_{a}	23.5%					0.398
**IFNγ**	3_{a}	2.5%	4_{a}	4.0%	3_{a}	4.4%					0.772
**IL-2**	19_{a}	15.7%	11_{a}	11.1%	10_{a}	14.7%					0.642
**IL-12p70**	3_{a}	2.5%	4_{a}	4.0%	3_{a}	4.4%					0.772
**IL-4**	22_{a}	18.2%	11_{a}	11.1%	14_{a}	20.6%					0.198
**IL-5**	11_{a}	9.1%	11_{a}	11.1%	13_{a}	19.1%					0.115
**IL-13**	22_{a}	18.2%	16_{a}	16.2%	11_{a}	16.2%					0.920
**IL-17A**	19_{a}	15.7%	7_{a}	7.1%	9_{a}	13.2%					0.151
**IL-17F**	36_{a}	29.8%	24_{a}	24.2%	17_{a}	25.0%					0.636
**IL-21**	13_{a}	10.7%	10_{a}	10.1%	5_{a}	7.4%					0.768
**IL-22**	11_{a}	10.9%	11_{a}	12.8%	8_{a}	12.9%					0.909
**IL-6**	4_{a}	3.3%	5_{a}	5.1%	2_{a}	2.9%					0.732
**IL-9**	15_{a}	12.4%	12_{a}	12.1%	13_{a}	19.1%					0.373
**GM-CSF**	3_{a}	2.5%	6_{a}	6.1%	2_{a}	2.9%					0.387
**IL-10**	7_{a}	5.7%	10_{a}	10.1%	5_{a}	7.4%					0.454
**HIV+ women**											
**IL-1β**	2_{a}	3.7%	3_{a}	6.7%	3_{a}	9.7%	0[1]	0.0%	0[1]	0.0%	0.617
**TNF-α**	1_{a}	1.9%	3_{a}	6.7%	2_{a}	6.5%	0[1]	0.0%	0[1]	0.0%	0.613
**IP-10**	54[1]	100.0%	45[1]	100.0%	31[1]	100.0%	11[1]	100.0%	18[1]	100.0%	--
**IL-8**	2_{a}	3.7%	3_{a}	6.7%	0[1]	0.0%	0[1]	0.0%	0[1]	0.0%	0.603
**IFNα2**	3_{a}	5.6%	3_{a}	6.7%	5_{a}	16.1%	2_{a}	18.2%	0[1]	0.0%	0.162
**IFNβ**	7_{a}	13.0%	6_{a}	13.3%	3_{a}	9.7%	1_{a}	9.1%	0[1]	0.0%	0.614
**IFNλ1**	5_{a}	9.3%	21_{b}	46.7%	17_{b}	54.8%	$1_{a,b}$	9.1%	0[1]	0.0%	**0.000**
**IFNλ2/3**	4_{a}	7.4%	7_{a}	15.6%	2_{a}	6.5%	3_{a}	27.3%	0[1]	0.0%	0.097
**IFNγ**	0[1]	0.0%	1_{a}	2.2%	1_{a}	3.2%	0[1]	0.0%	0[1]	0.0%	0.559
**IL-2**	17_{a}	31.5%	9_{a}	20.0%	7_{a}	22.6%	4_{a}	36.4%	2_{a}	11.1%	0.345
**IL-12p70**	2_{a}	3.7%	4_{a}	8.9%	3_{a}	9.7%	1_{a}	9.1%	0[1]	0.0%	0.489
**IL-4**	11_{a}	20.4%	6_{a}	13.3%	8_{a}	25.8%	2_{a}	18.2%	0[1]	0.0%	0.130
**IL-5**	9_{a}	16.7%	10_{a}	22.2%	10_{a}	32.3%	3_{a}	27.3%	1_{a}	5.6%	0.190
**IL-13**	9_{a}	16.7%	8_{a}	17.8%	6_{a}	19.4%	2_{a}	18.2%	1_{a}	5.6%	0.772
**IL-17A**	16_{a}	29.6%	6_{a}	13.3%	6_{a}	19.4%	5_{a}	45.5%	4_{a}	22.2%	0.132
**IL-17F**	20_{a}	37.0%	13_{a}	28.9%	8_{a}	25.8%	5_{a}	45.5%	3_{a}	16.7%	0.377
**IL-21**	3_{a}	5.6%	3_{a}	6.7%	2_{a}	6.5%	2_{a}	18.2%	0[1]	0.0%	0.443
**IL-22**	8_{a}	14.8%	7_{a}	15.6%	4_{a}	12.9%	4_{a}	36.4%	2_{a}	11.1%	0.460
**IL-6**	4_{a}	7.4%	4_{a}	8.9%	2_{a}	6.5%	1_{a}	9.1%	1_{a}	5.6%	1.000
**IL-9**	11_{a}	20.4%	6_{a}	13.3%	7_{a}	22.6%	2_{a}	18.2%	2_{a}	11.1%	0.771
**GM-CSF**	2_{a}	3.7%	2_{a}	4.4%	2_{a}	6.5%	0[1]	0.0%	0[1]	0.0%	0.924
**IL-10**	7_{a}	13.0%	7_{a}	15.6%	5_{a}	16.1%	3_{a}	27.3%	1_{a}	5.6%	0.584
**HIV- women**											
**IL-1β**	21_{a}	30.9%	13_{a}	24.1%	6_{a}	16.2%					0.245

**TNF-α**	0[1]	0.0%	1_a	1.9%	0[1]	0.0%	0.572
**IP-10**	29_a	42.6%	37_b	68.5%	26_b	70.3%	**0.003**
**IL-8**	2_a	2.9%	3_a	5.6%	2_a	5.4%	0.693
**IFNα2**	3_a	4.4%	4_a	7.4%	2_a	5.4%	0.905
**IFNβ**	29_a	42.6%	20_a	37.0%	12_a	32.4%	0.567
**IFNλ1**	7_a	10.3%	12_a	22.2%	8_a	21.6%	0.152
**IFNλ2/3**	31_a	45.6%	26_a	48.1%	14_a	37.8%	0.647
**IFNγ**	3_a	4.4%	3_a	5.6%	2_a	5.4%	1.000
**IL-2**	2_a	3.0%	2_a	3.7%	3_a	8.1%	0.483
**IL-12p70**	1_a	1.5%	0[1]	0.0%	0[1]	0.0%	1.000
**IL-4**	11_a	16.4%	5_a	9.3%	6_a	16.2%	0.472
**IL-5**	2_a	3.0%	1_a	1.9%	3_a	8.1%	0.321
**IL-13**	13_a	19.4%	8_a	14.8%	5_a	13.5%	0.733
**IL-17A**	3_a	4.5%	1_a	1.9%	3_a	8.1%	0.426
**IL-17F**	16_a	23.9%	11_a	20.4%	9_a	24.3%	0.885
**IL-21**	10_a	14.9%	7_a	13.0%	3_a	8.1%	0.620
**IL-22**	3_a	6.4%	4_a	9.8%	4_a	12.9%	0.567
**IL-6**	0[1]	0.0%	1_a	1.9%	0[1]	0.0%	0.572
**IL-9**	4_a	6.0%	6_a	11.1%	6_a	16.2%	0.256
**GM-CSF**	1_a	1.5%	4_a	7.4%	0[1]	0.0%	0.127
**IL-10**	0[1]	0.0%	3_a	5.6%	0[1]	0.0%	0.050

Supplementary Table 1. Systemic cytokines across pregnancy. Cytokines and chemokines were measured in maternal plasma collected during the first, second and third trimester in all women studied (both women living with HIV (HIV+ women) and HIV-negative women (HIV- women)), as well as at delivery and six weeks postnatal for 11 and 18 women living with HIV, respectively. Data presented are the number and percentage of samples in which cytokines were detected by multiplex cytokine assay. The Chi-square test or Fisher's exact test was used for statistical comparisons as appropriate. Values in the same row not sharing the same subscript are significantly different in the two-sided test of equality for column proportions. Cells with no subscript are not included in the test. [1]This category is not used in comparisons because its column proportion is equal to zero or one.

		HIV Status					Preterm Status					SGA Status				
		Positive		Negative			Preterm		Term			SGA		no SGA		
		n		n		*P*	n		n		*P*	n		n		*P*
**IL-1β**	T1	2	3.7%	21	30.9%	**0.000**	12	25.5%	11	14.7%	0.158	6	20.7%	17	18.3%	0.789
	T2	3	6.7%	13	24.1%	**0.027**	6	19.4%	10	14.7%	0.770	5	21.7%	11	14.5%	0.518
	T3	3	9.7%	6	16.2%	0.494	0	0.0%	9	13.6%	1.000	4	22.2%	5	10.0%	0.231
**TNF-α**	T1	1	1.9%	0	0.0%	0.443	0	0.0%	1	1.3%	1.000	1	3.4%	0	0.0%	0.238
	T2	3	6.7%	1	1.9%	0.327	1	3.2%	3	4.4%	1.000	2	8.7%	2	2.6%	0.230
	T3	2	6.5%	0	0.0%	0.204	0	0.0%	2	3.0%	1.000	1	5.6%	1	2.0%	0.462
**IP-10**	T1	54	100.0%	29	42.6%	**0.000**	28	59.6%	55	73.3%	0.162	22	75.9%	61	65.6%	0.366
	T2	45	100.0%	37	68.5%	**0.000**	25	80.6%	57	83.8%	0.776	20	87.0%	62	81.6%	0.755
	T3	31	100.0%	26	70.3%	**0.001**	2	100.0%	55	83.3%	1.000	14	77.8%	43	86.0%	0.464
**IL-8**	T1	2	3.7%	2	2.9%	1.000	1	2.1%	3	4.0%	1.000	2	6.9%	2	2.2%	0.239
	T2	3	6.7%	3	5.6%	1.000	3	9.7%	3	4.4%	0.374	2	8.7%	4	5.3%	0.621
	T3	0	0.0%	2	5.4%	0.496	0	0.0%	2	3.0%	1.000	0	0.0%	2	4.0%	1.000
**IFNα2**	T1	3	5.6%	3	4.4%	1.000	0	0.0%	6	8.0%	0.081	1	3.4%	5	5.4%	1.000
	T2	3	6.7%	4	7.4%	1.000	2	6.5%	5	7.4%	1.000	3	13.0%	4	5.3%	0.348
	T3	5	16.1%	2	5.4%	0.233	1	50.0%	6	9.1%	0.197	2	11.1%	5	10.0%	1.000
**IFNβ**	T1	7	13.0%	29	42.6%	**0.001**	19	40.4%	17	22.7%	**0.043**	6	20.7%	30	32.3%	0.233
	T2	6	13.3%	20	37.0%	**0.011**	9	29.0%	17	25.0%	0.806	6	26.1%	20	26.3%	1.000
	T3	3	9.7%	12	32.4%	**0.038**	0	0.0%	15	22.7%	1.000	4	22.2%	11	22.0%	1.000
**IFNλ1**	T1	5	9.3%	7	10.3%	1.000	2	4.3%	10	13.3%	0.127	3	10.3%	9	9.7%	1.000
	T2	21	46.7%	12	22.2%	**0.018**	11	35.5%	22	32.4%	0.820	10	43.5%	23	30.3%	0.313
	T3	17	54.8%	8	21.6%	**0.006**	1	50.0%	24	36.4%	1.000	8	44.4%	17	34.0%	0.570
**IFNλ2/3**	T1	4	7.4%	31	45.6%	**0.000**	14	29.8%	21	28.0%	0.840	8	27.6%	27	29.0%	1.000
	T2	7	15.6%	26	48.1%	**0.001**	9	29.0%	24	35.3%	0.648	7	30.4%	26	34.2%	0.805
	T3	2	6.5%	14	37.8%	**0.003**	0	0.0%	16	24.2%	1.000	5	27.8%	11	22.0%	0.747
**IFNγ**	T1	0	0.0%	3	4.4%	0.254	1	2.1%	2	2.7%	1.000	0	0.0%	3	3.2%	1.000
	T2	1	2.2%	3	5.6%	0.624	1	3.2%	3	4.4%	1.000	1	4.3%	3	3.9%	1.000
	T3	1	3.2%	2	5.4%	1.000	0	0.0%	3	4.5%	1.000	1	5.6%	2	4.0%	1.000
**IL-2**	T1	17	31.5%	2	3.0%	**0.000**	8	17.4%	11	14.7%	0.798	8	27.6%	11	12.0%	0.075
	T2	9	20.0%	2	3.7%	**0.021**	2	6.5%	9	13.2%	0.494	5	21.7%	6	7.9%	0.121
	T3	7	22.6%	3	8.1%	0.167	0	0.0%	10	15.2%	1.000	3	16.7%	7	14.0%	0.717
**IL-12p70**	T1	2	3.7%	1	1.5%	0.583	0	0.0%	3	4.0%	0.284	1	3.4%	2	2.2%	0.560
	T2	4	8.9%	0	0.0%	**0.040**	2	6.5%	2	2.9%	0.587	2	8.7%	2	2.6%	0.230
	T3	3	9.7%	0	0.0%	0.090	0	0.0%	3	4.5%	1.000	1	5.6%	2	4.0%	1.000
**IL-4**	T1	11	20.4%	11	16.4%	0.639	8	17.4%	14	18.7%	1.000	9	31.0%	13	14.1%	0.053
	T2	6	13.3%	5	9.3%	0.750	4	12.9%	7	10.3%	0.736	4	17.4%	7	9.2%	0.275
	T3	8	25.8%	6	16.2%	0.378	0	0.0%	14	21.2%	1.000	3	16.7%	11	22.0%	0.745
**IL-5**	T1	9	16.7%	2	3.0%	**0.012**	3	6.5%	8	10.7%	0.530	5	17.2%	6	6.5%	0.131
	T2	10	22.2%	1	1.9%	**0.002**	5	16.1%	6	8.8%	0.313	5	21.7%	6	7.9%	0.121
	T3	10	32.3%	3	8.1%	**0.015**	0	0.0%	13	19.7%	1.000	6	33.3%	7	14.0%	0.090
**IL-13**	T1	9	16.7%	13	19.4%	0.814	8	17.4%	14	18.7%	1.000	10	34.5%	12	13.0%	**0.014**
	T2	8	17.8%	8	14.8%	0.787	5	16.1%	11	16.2%	1.000	5	21.7%	11	14.5%	0.518
	T3	6	19.4%	5	13.5%	0.742	0	0.0%	11	16.7%	1.000	5	27.8%	6	12.0%	0.144

**IL-17A**	T1	16	29.6%	3	4.5%	**0.000**	10	21.7%	9	12.0%	0.199	7	24.1%	12	13.0%	0.157
	T2	6	13.3%	1	1.9%	**0.044**	1	3.2%	6	8.8%	0.429	3	13.0%	4	5.3%	0.348
	T3	6	19.4%	3	8.1%	0.282	0	0.0%	9	13.6%	1.000	1	5.6%	8	16.0%	0.427
**IL-17F**	T1	20	37.0%	16	23.9%	0.161	17	37.0%	19	25.3%	0.220	14	48.3%	22	23.9%	**0.019**
	T2	13	28.9%	11	20.4%	0.355	8	25.8%	16	23.5%	1.000	9	39.1%	15	19.7%	0.093
	T3	8	25.8%	9	24.3%	1.000	0	0.0%	17	25.8%	1.000	7	38.9%	10	20.0%	0.126
**IL-21**	T1	3	5.6%	10	14.9%	0.141	5	10.9%	8	10.7%	1.000	4	13.8%	9	9.8%	0.509
	T2	3	6.7%	7	13.0%	0.340	5	16.1%	5	7.4%	0.279	3	13.0%	7	9.2%	0.694
	T3	2	6.5%	3	8.1%	1.000	0	0.0%	5	7.6%	1.000	2	11.1%	3	6.0%	0.602
**IL-22**	T1	8	14.8%	3	6.4%	0.213	5	14.3%	6	9.1%	0.507	4	16.7%	7	9.1%	0.286
	T2	7	15.6%	4	9.8%	0.526	0	0.0%	11	18.3%	**0.030**	4	20.0%	7	10.6%	0.272
	T3	4	12.9%	4	12.9%	1.000	0	0.0%	8	13.3%	1.000	2	13.3%	6	12.8%	1.000
**IL-6**	T1	4	7.4%	0	0.0%	**0.036**	0	0.0%	4	5.3%	0.159	1	3.4%	3	3.2%	1.000
	T2	4	8.9%	1	1.9%	0.174	2	6.5%	3	4.4%	0.647	3	13.0%	2	2.6%	0.08
	T3	2	6.5%	0	0.0%	0.204	0	0.0%	2	3.0%	1.000	1	5.6%	1	2.0%	0.462
**IL-9**	T1	11	20.4%	4	6.0%	**0.025**	3	6.5%	12	16.0%	0.161	7	24.1%	8	8.7%	**0.048**
	T2	6	13.3%	6	11.1%	0.766	2	6.5%	10	14.7%	0.331	3	13.0%	9	11.8%	1.000
	T3	7	22.6%	6	16.2%	0.549	0	0.0%	13	19.7%	1.000	3	16.7%	10	20.0%	1.000
**GM-CSF**	T1	2	3.7%	1	1.5%	0.583	2	4.3%	1	1.3%	0.558	0	0.0%	3	3.2%	1.000
	T2	2	4.4%	4	7.4%	0.686	1	3.2%	5	7.4%	0.662	2	8.7%	4	5.3%	0.621
	T3	2	6.5%	0	0.0%	0.204	0	0.0%	2	3.0%	1.000	0	0.0%	2	4.0%	1.000
**IL-10**	T1	7	13.0%	0	0.0%	**0.003**	1	2.1%	6	8.0%	0.247	3	10.3%	4	4.3%	0.355
	T2	7	15.6%	3	5.6%	0.178	4	12.9%	6	8.8%	0.720	3	13.0%	7	9.2%	0.694
	T3	5	16.1%	0	0.0%	**0.016**	0	0.0%	5	7.6%	1.000	1	5.6%	4	8.0%	1.000

Supplementary Table 2. Systemic cytokines across pregnancy according to HIV status, preterm birth status and small-for-gestational-age (SGA) status. Cytokines and chemokines were measured in maternal plasma collected during the first, second and third trimester. Data presented are the number and percentage of samples in which cytokines were detected by multiplex cytokine assay. The Chi-square test or Fisher's exact test was used for statistical comparisons as appropriate.

	Trimester										
	1		2		3		Delivery		Postnatal		*P*
	n		n		n		n		n		
**All Women**											
**Pro-inflammatory**	101	82.8%	92	92.9%	61	89.7%					0.062
**Anti-viral**	46	37.7%	55	55.6%	98	56.7%					**0.009**
**Th1**	22	18.0%	17	17.2%	13	19.1%					0.950
**Th2**	31	25.6%	28	28.8%	23	33.8%					0.486
**Th17**	47	38.8%	37	37.4%	21	30.9%					0.537
**Pleiotropic**	20	16.4%	19	19.2%	14	20.6%					0.746
**HIV+ women**											
**Pro-inflammatory**	54	100%	45	100%	31	100%	11	100%	18	100%	--
**Anti-viral**	13	24.1%	25	55.6%	22	71.0%	3	27.3%	0	0.0%	**<0.0001**
**Th1**	17	31.5%	12	26.7%	8	25.8%	4	36.4%	2	11.1%	0.499
**Th2**	13	24.1%	16	35.6%	14	45.2%	3	27.3%	1	5.6%	**0.037**
**Th17**	22	40.7%	15	33.3%	9	29.0%	5	45.5%	6	33.3%	0.772
**Pleiotropic**	15	27.8%	10	22.2%	8	25.8%	3	27.3%	3	16.7%	0.893
**HIV- women**											
**Pro-inflammatory**	47	69.1%	47	87.0%	30	81.1%					0.052
**Anti-viral**	33	48.5%	30	55.6%	16	44.4%					0.557
**Th1**	5	7.4%	5	9.3%	5	13.5%					0.587
**Th2**	18	26.9%	12	22.2%	9	24.3%					0.839
**Th17**	25	37.3%	22	40.7%	12	32.4%					0.723
**Pleiotropic**	5	7.4%	9	16.7%	6	16.2%					0.228

Supplementary Table 3. Systemic cytokine subgroups across pregnancy. Cytokines and chemokines were measured in maternal plasma collected during the first, second and third trimester in all women studied (both women living with HIV (HIV+ women) and HIV-negative women (HIV- women)), as well as at delivery and six weeks postnatal for 11 and 18 women living with HIV, respectively. Data presented are the number and percentage of samples in which subgroups of cytokines were detected, i.e. pro-inflammatory (IL-1β, TNF-α, IP-10, IL-8), anti-viral (IFNα2, IFNβ, IFNλ1, IFNλ2/3), Th1 (IFNγ, IL-2, IL-12p70), Th2 (IL-4, IL-5, IL-13), Th17 (IL-17A, IL-17F, IL-21, IL-22) and pleiotropic (IL-6, IL-9, GM-CSF, IL-10) cytokines. The Chi-square test or Fisher's exact test was used for statistical comparisons as appropriate.

		HIV Status					Preterm Status					SGA Status				
		Positive		Negative			Preterm		Term			SGA		no SGA		
		**n**		**n**		***P***	**n**		**n**		***P***	**n**		**n**		***P***
**Pro-inflammatory**	T1	54	100%	47	69.1%	**<0.0001**	38	80.9%	63	84.0%	0.654	26	89.7%	60	76.9%	0.178
	T2	45	100%	47	87.0%	**0.015**	29	93.6%	63	92.7%	>0.99	22	95.7%	56	90.3%	0.668
	T3	31	100%	30	81.1%	**0.013**	2	100%	59	89.4%	>0.99	16	88.9%	35	87.5%	>0.99
**Anti-viral**	T1	13	24.1%	33	48.5%	**0.006**	20	42.6%	26	34.7%	0.382	9	31.0%	28	33.3%	0.820
	T2	25	55.6%	24	55.6%	>0.99	19	61.3%	36	52.9%	0.438	14	60.9%	33	48.5%	0.306
	T3	22	71.0%	16	44.4%	**0.029**	2	100%	36	55.4%	0.502	12	66.7%	20	46.5%	0.151
**Th1**	T1	17	31.5%	5	7.4%	**0.0006**	9	19.2%	13	17.3	0.800	8	27.6%	12	13.2%	0.070
	T2	12	26.7%	5	9.3%	**0.022**	5	16.1%	12	17.7%	0.853	6	26.1%	10	13.3%	0.148
	T3	8	25.8%	5	13.5%	0.199	0	0.0%	13	19.7%	>0.99	4	22.2%	7	14.6%	0.474
**Th2**	T1	13	24.1%	18	26.9%	0.727	12	26.1%	19	25.3%	0.927	12	41.4%	16	18.0%	**0.010**
	T2	16	35.6%	12	22.2%	0.143	10	32.3%	18	26.5%	0.553	10	43.5%	13	18.3%	**0.015**
	T3	14	45.2%	9	24.3%	0.071	0	0.0%	23	34.9%	0.546	8	44.4%	12	25.5%	0.139
**Th17**	T1	22	40.7%	25	37.3%	0.701	23	50.0%	24	32.0%	**0.049**	16	55.2%	24	28.2%	**0.009**
	T2	15	33.3%	22	40.7%	0.448	13	41.9%	24	35.3%	0.526	11	47.8%	19	27.5%	0.072
	T3	9	42.9%	12	57.1%	0.763	0	0.0%	21	31.8%	>0.99	8	44.4%	11	22.9%	0.085
**Pleiotropic**	T1	15	27.8%	5	7.4%	**0.003**	5	10.6%	15	20.0%	0.174	7	24.1%	10	11.1%	0.081
	T2	10	22.2%	9	16.7%	0.485	5	16.1%	14	20.6%	0.601	6	26.1%	8	11.3%	0.083
	T3	8	25.8%	6	16.2%	0.330	0	0.0%	14	21.2%	>0.99	3	16.7%	8	17.0%	>0.99

Supplementary Table 4. Systemic cytokine subgroups across pregnancy according to HIV status, preterm birth status and small-for-gestational-age (SGA) status. Cytokines and chemokines were measured in maternal plasma collected during the first, second and third trimester. Data presented are the number and percentage of samples in which subgroups of cytokines were detected, i.e. pro-inflammatory (IL-1β, TNF-α, IP-10, IL-8), anti-viral (IFNα2, IFNβ, IFNλ1, IFNλ2/3), Th1 (IFNγ, IL-2, IL-12p70), Th2 (IL-4, IL-5, IL-13), Th17 (IL-17A, IL-17F, IL-21, IL-22) and pleiotropic (IL-6, IL-9, GM-CSF, IL-10) cytokines. The Chi-square test or Fisher's exact test was used for statistical comparisons as appropriate.
